# Neurodegeneration with Brain Iron Accumulation: An Overview

**Published:** 2014

**Authors:** Seyed Hassan TONEKABONI, Mohsen MOLLAMOHAMMADI

**Affiliations:** 1 Pediatric Neurology Research Center, Shahid Beheshti University of Medical Sciences (SBMU), Tehran, Iran; 2 Pediatric Neurology Center of Excellence, Department of Pediatric Neurology, Mofid Children Hospital, Faculty of Medicine, Shahid Beheshti University of Medical Sciences (SBMU), Tehran, Iran; 3 Pediatric Neurology Department, Hazrat Fatemeh Masoumeh Hospital, Qom University of Medical Sciences, Qom, Iran

**Keywords:** Pantothenate Kinase Associated Neurodegeneration, Neurodegeneration With Brain Iron Accumulation, Hallervorden- Spatz syndrome, Neuroaxonal Dystrophy

## Abstract

Neurodegeneration with brain iron accumulation (NBIA) is a group of neurodegenerative disorder with deposition of iron in the brain (mainly Basal Ganglia) leading to a progressive Parkinsonism, spasticity, dystonia, retinal degeneration, optic atrophy often accompanied by psychiatric manifestations and cognitive decline. 8 of the 10 genetically defined NBIA types are inherited as autosomal recessive and the remaining two by autosomal dominant and X-linked dominant manner. Brain MRI findings are almost specific and show abnormal brain iron deposition in basal ganglia some other related anatomical locations. In some types of NBIA cerebellar atrophy is the major finding in MRI.

## Introduction

Deposition of Iron in brain causes a spectrum of disorders characterized by a progressive neurodegeneration and extrapyramidal symptoms. This brain Iron accumulation was first reported by Julius Hallervorden and Hugo Spatz, German neuropathologist, who conducted studies on brain specimens of mentally handicapped persons, executed during third Reich euthanasia program in the context of “Racial Hygiene”(1).

**Fig 1:**
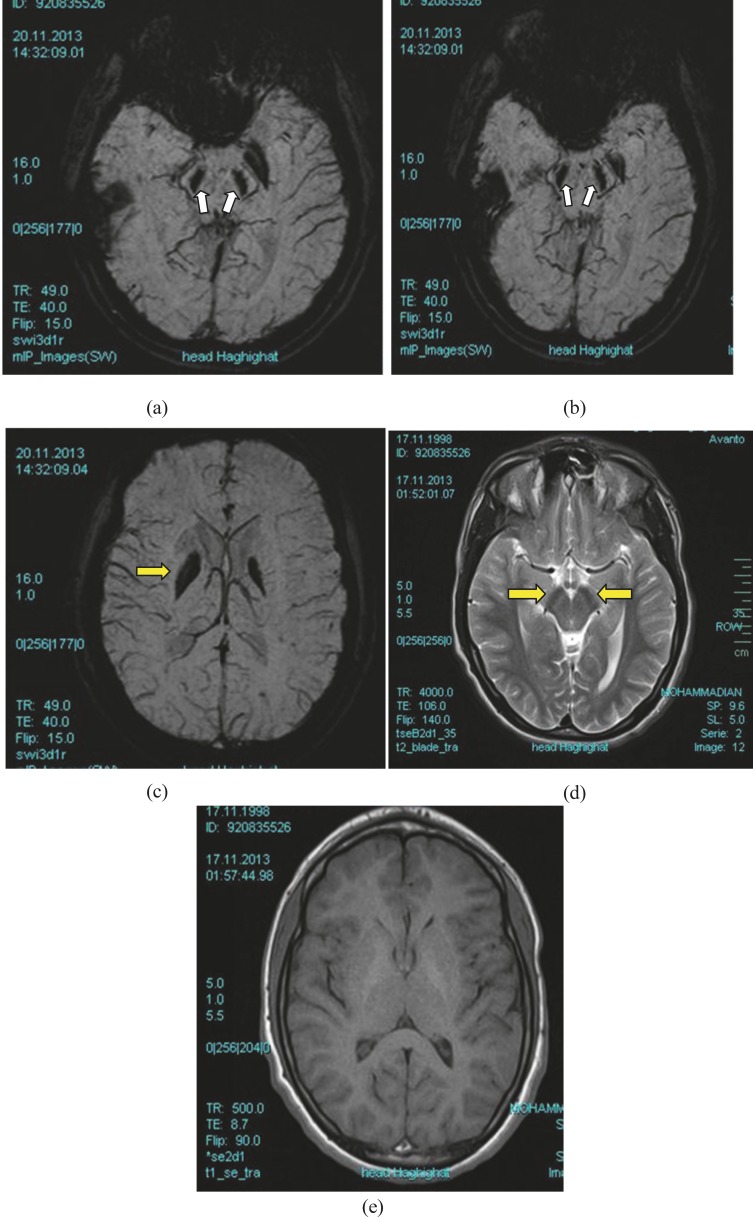
A 12 year-old boy with progressive dystonia-Parkinsonism & cognitive decline since early childhood (a, b):SWI sequence which is tailored to detect Iron deposition represents signal void Iron in the Substantial Nigra & Brain Stem (white arrows) apparently which is not detectable in other sequences. (c): Signal void abnormality as black part in SWI sequence in the bilateral BG confirming Iron deposition disorder (yellow arrow). (d): This is Axial T2W sequence and represents reduced signal due to Iron deposition in Substantial Nigra which is not well detectable. (Yellow arrows). (e): Pre and post contrast T1W represent no abnormal enhancement. No define finding related to Iron deposition is seen in the sequence. (f 1&2): In the Flair sequence there is signal void appearance and reduced signal pattern in Globus Pallidus (f1) which may be missed easily: in this sequence Iron deposition in the Substantial Nigra is not detectable (f2).

Today, this Iron deposition is easily diagnosed by magnetic resonance imaging techniques (figure 1). The classic form of Hallervorden-Spatz syndrome consisted of progressive gait and postural difficulties since early childhood. This was followed by other extra pyramidal signs like Parkinsonism and also cognitive decline characterized by behavioral changes and dementia (2). When a novel Pantothenate Kinase gene (PANK2) was identified as being defective inHallervorden-Spatz syndrome, and regarding serious ethical issues in the scientific activities of the two German neuropathologist, a new nomenclature was suggested. So this syndrome was named Pantothenate-Kinase associated neurodegeneration (PKAN). However, recent advances in brain MRI modalities and genetic linkage techniques led us to recognitionof broader spectrum phenotypes in a group of syndromes having in common: Iron accumulation in the brain, mainly in basal ganglia (preferentially the globus pallidus) and a clinical neurodegenerative course with prominent extra pyramidal symptoms. These are named syndromes of Neurodegeneration with Brain Iron Accumulation (NBIA) (3).

By now ten established genes, with their causative mutations, are known to cause different sub types of NBIA (table 1) (4). Although NBIA syndromes are rare genetic disorders with classic Mendelian inheritance (mostly autosomal Recessive type), some pathologic mutations in NBIA genes have been reported in rare monogenic Mendelian inheritance cases of Parkinson disease like ATP13A2 and PLA2G6 gene that are assigned respectively as PARK9 and PARK14 gene. Adding to this, demonstration of Lewy bodies and tau-positive tangles in brain specimens of NBIA cases (a pathologic finding in some adult onset sporadic neurodegenerative disease including Parkinson, Alzheimer and Fronto temporal dementia), shows an overlap between more common neurodegenerative disorders and some of the NBIA syndromes (5). There is also an overlap with other genetic diseases, once thought as being distinct conditions and usually not associated with Iron accumulation, as an example, mutations in FA2H gene lead to a FAHN phenotype (of NBIA group) while mutation in the same gene causes a form of hereditary spastic paraplegia (HSP) (6) and a leukodystrophy with spastic paraparesis and dystonia (7). Except aceruloplasminemia and neuroferritinopathy, mutations in other genes implicated in NBIA, are not directly involved in metabolic pathways of iron metabolism. Although demonstration of iron accumulation in the brain is essential for diagnosis of NBIA, however we still don’t know if iron deposition is the underlying key pathologic process in NBIA or if it is only an epiphenomenon of neurodegeneration.

NBIA is a clinicoradiological entity characterized by progressive neurological deterioration with prominent extrapyramidal symptoms, intellectual impairment and iron accumulation identified on brain MRI. There is significant phenotypic heterogeneity among NBIA syndromes and also an overlap with other genetic neurodegenerative disorders (spinocerebellar ataxia, Hereditary spastic paraplegia, Leukodystrophy, hereditary dystonia and mitochondrial disorder). 

**NBIA type 1: PKAN**

Pantothenate Kinase-Associated Neurodegeneration (PKAN) is the most common and the most studied disorder of the NBIA group. In medical literature it was formerly known as Hallervorden-Spatz and also is the major component of the neuroaxonal dystrophies group. PKAN has variable phenotype which is mainly age dependent: the classic presentation has early onset and the atypical one (non-classic form) has later onset manifestation.

In the classic form ataxia and postural difficulties start in early childhood. This is followed by progressive extra pyramidal symptoms: dystonia, chorea and Parkinsonism (2). Severe tongue protrusion dystonia is a prominent sign and along with limb dystonia causes severe disability in motor function within few years of disease onset (8). This motor regression is accompanied by cognitive decline and dementia. Impaired saccadic pursuit eye movements and disturbed vertical saccades (which is a major sign in Niemann-Pick C disease) herald mid-brain degeneration. Other ophthalmologic pathology consists of pigmentary retinopathy in 40% of patients, abnormal electroretinogram in two third and pupillary abnormalities (9). Pigmentary retinopathy is strongly in favor of PKAN and is absent in other forms of NBIA. But optic atrophy can also appear in FAHN, PLAN and MPAN.

Atypical (late onset) PKAN’s are less common than classic form and mostly unrecognized regarding nonspecific neurologic presentations in adulthood. These symptoms consist of extrapyramidal complains like focal dystonia, unilateral tremor, retinopathy, cognitive deterioration, speech abnormality and psychiatric symptoms (for example palilalia) (3). Generally dyskinetic symptoms are milder in late onset patients when compared to classic PKAN and they remain ambulatory for decades. PKAN has a characteristic brain MRI pattern called “eye of the tiger sign” and consists of bilateral hyper intensity within surrounding hypo intensity in the globuspallidus in T2-weighted images. Pathologically there is a mild simultaneous involvement of substantia nigra pars reticulata, which may or may not appear on brain MRI. Practically, when such lesions are detected in T2-weighted images of a patient with clinical presentation compatible with PKAN, gradient-echo T2 and susceptibility weighted imaging sequences should be reviewed to confirm iron accumulation. Other disorder showing signal changes of the basal ganglia such as organic acidemia, disorder of cofactor metabolism and mitochondropathies feature T2 hyper intensity rather than hypo intensity (10). The eye of the tiger sign can be found in presymptomatic patients with PKAN and the central hyper intensity of the Globus pallidus can fade with disease progression, showing the dynamic nature of MRI findings in PKAN. 

PKAN is an autosomal recessive disease with mutations detected in all seven exons of the PANK2 gene. Mutations are mostly missense; however duplication, deletion, splice-site mutation and exon deletion have also been reported (11, 12). It is still unresolved how mutation in PANK2 gene leads to iron accumulation. The protein encoded by PANK2 gene has a pivotal role in the metabolism of the vitamin B5 (pantothenate) (13). It has been hypothesized that lipids may be required for regulation of iron transport proteins and the alternation of ferroprotein expression, which is mediated by PANK2 gene, may lead to iron brain deposition (14). Previous reports of Lewy body pathology and α-synuclein in PKAN (15,16) was before the identification of PANK2 gene, and recent studies in genetically confirmed cases of PKAN contradict these results and no such pathology was found (17). Probably some of historical patients with Hallervorden-Spatz syndrome with α-synuclein accumulation and Lewy body pathology had not PKAN and they suffered from other NBIA.

**NBIA type2: PLAN**

Phospo-Lipase-Associated Neurolodegeneration(PLAN) is the second core of NBIA syndromes. Like PKAN there is age related phenotype variability with a clinical spectrum from infancy (early-onset) to adulthood (late-onset).in early onset cases affected children have progressive loss of cognitive and motor function. It is characterized by marked axial hypotonia, cerebellar ataxia, four limb spasticity, strabismus and bulbar dysfunction. Optic atrophy is a common finding and seizure disorder is noticed in some patients, although EEG changes as fast activity is a more frequent finding (18). Cerebellar cortical atrophy and gliosis is the most common finding in brain MRI. Late onset PLAN is characterized by a dystonia- Parkinsonism syndrome accompanied by pyramidal signs starting in adulthood.

Abnormal eye movements, dementia and psychiatric manifestation may coexist. Parkinsonism is presented by tremor (pill rolling at rest), bradykinesia and rigidity with a favorable response to levodopa. In contrast to PKAN patients, Brain specimens in Plans show the alpha-synuclein-positive Lewy body pathology (19).

Cerebellar signs are less observed in late onset PLAN in contrast to dyskinesia which is a frequent finding. This fact is reinforced by the observation of cerebellar atrophy in early onset PLAN which is not a typical feature of the late onset form. Iron accumulation in Globus Pallidus causes hypointensity in MRI, but the “eye of the tiger sign” is not seen because of lack central hyperintensity. 

Brain MRI may be normal without any iron deposition, thus all patients with PLAN are not included in the larger group of NBIA.

Mutations PLA2G6 gene cause PLAN. This gene encodes the protein iPLA2 beta, which is involved in free fatty acids and lysophospholipidssynthesis. Disturbance of fatty acid metabolism can result in membrane trafficking dysregulation and in line with their accumulation, progressive neurologic deterioration supervene. PLA2G6 gene is a candidate gene for one form of monogenic Parkinson disease and has been assigned as ParK14 locus.

**MPAN (Mitochondrial Protein Associated Neurodegeneration):**

Mutation in the orphan gene C19orf12 (MMIN) is responsible for MPAN. Because of mitochondrial localization of tagged C19orf12 protein, dysfunction of fatty acids in the mitochondria is hypothesized as the pathologic mechanism of this disorder. C19orf12 is located on chromosome 19q12 and is not part of mitochondrial genome. In the largest studied group in Poland, clinical manifestations started in childhood with speech and gait disturbance followed by pyramidal – extrapyramidal signs, psychiatric symptoms and optic atrophy (20). Dystonia and prakinsonism were the major movement disorder observed in these patients. Iron accumulation was detected in Globus Pallidusand Substantia Nigra.

**FAHN (Fatty Acid Hydroxylase-associated Neurodegeneration):**

FAHN symptoms typically start in childhood with slowly progressive gait disturbance and focal dystonia in the legs and feet. Spastic quadriparesis, ataxia, dysarthria and progressive visual loss due to optic atrophy develop later. Loss of cognitive function is reported in most of the patients and seizure disorder may be present in some. Neuroimaging reveals bilateral T2 hypointensity in Globus Pallidus, consistent with iron accumulation. White matter changes with or without brain stem and cerebellar atrophy increase gradually and may become profound.

The disease is caused by mutation in Fatty acid-2 Hydroxylase (FA2H) gene which is involved in lipid and ceramid metabolic pathway. Mutations in this gene are responsible for two other overlapping syndromes: one with leukodystrophy manifestations and the other with hereditary spastic paraplegia phenotype.

**Kufor- Rakeb disease**

The disease was first reported in a Jordanian family as a syndrome with pallido-pyramidal degeneration, supra nuclear upgaze paresis and dementia (21). Mutations in ATP13A2 gene, encoding a lysosomal type 5 P-type ATPase is known to be responsible for the clinical manifestations.

Kufor –Rakeb syndrome is characterized by juvenile onset Levodopa_responsive parkinsonism, pyramidal tract sign, supra nuclear gaze palsy (accompanied by slowing of vertical and horizontal saccads and pursuit eye movement), oculogyric dystonic spasm, facialfaucial- finger myoclonus, visual hallucination and dementia.

Brain MRI shows moderate diffuse cerebral and cerebellar atrophy. Iron accumulation in caudate and Putamen is present in only a portion of patients, and may occur late in disease course. Kufor- Rakeb disease is also called PARK9 or Parkinson disease 9.

**BPAN (Beta- Propeller Protein- Associated Neurodegeneration):**

Affected individuals show a regression in motor, gait (with a dystonia-parkinsonism syndrome) and cognitive function in adulthood on a background of global developmental delay in childhood and adolescence (22). Mutations in WDR45 gene, located on X chromosome Xp11-23, are responsible for BPAN disease with a distinctive X-linked dominant mode of inheritance. This syndrome fulfills the criteria of the unidentified syndrome of SENDA (Static Encephalopathy of childhood with Neuro Degeneration in Adulthood) and probably previous reported cases suffered from BPAN. 

SENDA patients have intellectual disability and motor delay in infancy and childhood with slow progression in motor and intellectual function for one to third decades, when a dystonia-parkinsonism degenerative syndrome appears in adulthood.

**Neuroferritinopathy**

Neuroferritinopathy is an autosomal dominant inherited disorder (an exception in NBIA) due to mutation in FTL gene. Progressive chorea and dystonia appears in adulthood with mild cognitive decline. Dystonia starts from one limb and evolves into involvement of all limbs within 20 years. Orofacial action- specific dystonia is specific as well as orolingual dyskinesia (23). Cerebellar dysfunction can be seen in some families. Subtle dementia is present in most individuals which eventually becomes a major problem. Supranuclear gaze palsy may appear in some patients and in contrast to many other NBIA no ophthalmologic pathology is reported in this disease.

Serum ferritin concentration is low in the majority of men and post-menopausal women. Brain MRI is characteristic for the disease and shows cystic changes in the basal ganglia (advanced cases) with evidence of brain iron deposition on T2 MRI in globus pallidus, putamen, caudate nucleus, substantia nigra and red nuclei (24).

**Aceruloplasminemia**

Aceruloplasminemia is the only NBIA with simultaneous iron accumulation in both the brain and viscera. The clinical triad of neurologic disease, retinal degeneration and diabetes mellitus start in adulthood and older ages. Neurologic manifestations include orofacial dystonia with blepharospasm and grimacing, dysarthria, chorea, tremor and gait ataxia (25). A microcytic hypochromic anemia is usually the presenting sign before the onset of diabetes mellitus or obvious neurologic sign. 

Aceruloplasminemia is an autosomal recessive disease caused by mutation in CP (CeruloPlasmin) gene. Ceruloplasmin carries the majority of the plasma copper and plays a pivotal role in iron mobilization from tissues. Iron accumulation in brain and viscera is caused by ceruloplasmin dysfunction. 

Serum ceruloplasmin is undetectable, serum iron and copper is low and in contrast serum ferritin level is elevated.

Abdominal MRI shows abnormal hypointensity in the liver and brain MRI iron deposition is confirmed by T1 and T2 hypointensity in striatum, thalamus and dentate nucleus.

**CoPAN (COASY protein-associated neurodegeneration)**

This newly diagnosed NBIA with autosomal recessive mode of inheritance is caused by mutation in COASY ( CO enzyme A Synthetase) gene (26). The encoding protein is a bifunctional enzyme that catalyzes the two last steps in Co A synthesis. Biosynthesis of Coenzyme A from pantothenic acid (vitamin B5) is an essential pathway in human cells.

In the affected patients symptoms began with progressive spasticity and dystonia from early childhood, followed by dysarthria, oromandibular dystonia, parkinsonism and axonal neuropathy. 

Cognitive decline and psychiatric manifestations as obsessive compulsive behavior occur in the course of the disease. Usually the patients show slow regression and have survived into their third decade.

Brain iron deposition in globus pallidus and substantia nigra is detected as hypo intensities on T2W images of MRI.

**Woodhouse-Sakati syndrome**

This rare autosomal recessive multi systemic disorder consists of hypogonadism, diabetes mellitus alopecia mental retardation and extrapyramidal syndrome. A founder mutation in C2orf37 gene was reported in 8 consanguineous family in Saudi Arabia affected by this syndrome (27).

Basal ganglia hypo intensities on T2W brain MRI are consistent with iron deposition. White matter abnormality is common.

**Treatment**

Current treatment in NBIA is primarily symptomatic for relief of spasticity, dystonia and other movement disorder. Commonly used drugs are Benzodiazepines, anticholinergic, Baclofen, Neuroleptics and L-dopa (especially in parkinsonism). 

Deep Brain Stimulation (DBS) causes mild improvement in dystonia severity and no definite result has been achieved by iron chelating agents (deferiprone for example) clinical trials.

**Table 1:** Types of NBIA: Molecular Genetics

Disease Name	Gene	% of NBIA Attributed to Mutations in This Gene	Inheritance
PKAN	PANK2	35%-50%	AR
PLAN	PLA2G6	20%	AR
MPAN	C19orf12	6%-10%	AR
**BPAN**	WDR45	1%-2%	XLD
FAHN	FA2H	Rare	AR
**Kufor-Rakeb syndrome**	ATP13A2	Rare	AR
Neuroferritinopathy	FTL	Rare	AD
Aceruloplasminemia	CP	Rare	AR
**Woodhouse-Sakati syndrome**	DCAF17	Rare	AR
**CoPAN**	COASY	Rare	AR

PKAN= Pantothenate Kinase-associated Neurodegeneration

PLAN= PLA2G6-associated neurodegeneration

MPAN= Mitochondrial Membrane Protein-Associated Neurodegeneration

BPAN = Beta-Propeller Protein-Associated Neurodegeneration

FAHN = Fatty Acid Hydroxylase-Associated Neurodegeneration

CoPAN = Coasy Protein-Associated Neurodegeneration
